# Novel RNA variants in colorectal cancers

**DOI:** 10.18632/oncotarget.5500

**Published:** 2015-10-12

**Authors:** Andreas M. Hoff, Bjarne Johannessen, Sharmini Alagaratnam, Sen Zhao, Torfinn Nome, Marthe Løvf, Anne C. Bakken, Merete Hektoen, Anita Sveen, Ragnhild A. Lothe, Rolf I. Skotheim

**Affiliations:** ^1^ Department of Molecular Oncology, Institute for Cancer Research, Oslo University Hospital-Norwegian Radium Hospital, Oslo, Norway; ^2^ KG Jebsen Colorectal Cancer Research Centre, Oslo University Hospital, Oslo, Norway; ^3^ Centre for Cancer Biomedicine, University of Oslo, Oslo, Norway

**Keywords:** colorectal cancer, fusion genes, transcript variants, RACE-seq, splicing

## Abstract

With an annual estimated incidence of 1.4 million, and a five-year survival rate of 60%, colorectal cancer (CRC) is a major clinical burden. To identify novel RNA variants in CRC, we analyzed exon-level microarray expression data from a cohort of 202 CRCs. We nominated 25 genes with increased expression of their 3′ parts in at least one cancer sample each. To efficiently investigate underlying transcript structures, we developed an approach using rapid amplification of cDNA ends followed by high throughput sequencing (RACE-seq). RACE products from the targeted genes in 23 CRC samples were pooled together and sequenced. We identified *VWA2*-*TCF7L2*, *DHX35*-*BPIFA2* and *CASZ1*-*MASP2* as private fusion events, and novel transcript structures for 17 of the 23 other candidate genes. The high-throughput approach facilitated identification of CRC specific RNA variants. These include a recurrent read-through fusion transcript between *KLK8* and *KLK7*, and a splice variant of *S100A2*. Both of these were overrepresented in CRC tissue and cell lines from external RNA-seq datasets.

## INTRODUCTION

Colorectal cancer (CRC) alone accounts for close to 10% of all cancer cases worldwide, and is a heavy burden on human health and economy. It has been estimated that in total 694,000 people died from CRC in 2012 [1]. Developing through several molecular pathways, CRC is a heterogeneous disease with an urgent need for biomarkers carrying diagnostic, prognostic and predictive information. The cancer transcriptome represents a complex collection of RNA molecules which reflect the expression program of cancer cells at a given time. Aberrant gene expression signatures have been successfully identified for subclasses of cancer [2, 3] and for prognostication [4]. However, few gene expression signatures have yet been implemented in the clinic, and there is a high demand for additional tools to stratify the heterogeneous patient population.

Alternative pre-mRNA splicing and core promoter usage can create cancer-specific transcripts [5, 6], which has also been identified in CRC [7–10]. In addition, fusion transcripts, chimeric RNA joined together from two individual genes as a consequence of chromosomal rearrangements or complex post-transcriptional processes can be highly cancer-specific. Fusion genes which result from chromosomal rearrangements have been shown to be pathognomonic for certain cancer types and are used routinely as diagnostic markers in hematological cancers and childhood sarcomas [11, 12]. More recently, with the advent of genome-scale technologies, fusion genes have been identified also in adult epithelial cancers. Although most of them are present only in small subsets of carcinomas, some are found to be highly recurrent, such as rearrangement of *TMPRSS2* and *ETS* transcription factor genes in more than half of all prostate cancers [13]. Fusion transcripts may also be expressed as a result of transcriptional mechanisms such as trans-splicing and read-through events for adjacent genes [14]. Proof of non-random expression of such fusion transcripts in normal tissue types with translation into chimeric proteins have been described [15]. Several reports have shown that such fusion transcripts have an impact on cancer biology, by regulating both replication and cell growth in cancer [16–19]. Chimeric mRNAs expressed in normal cells are sometimes overexpressed in cancer cells. This is the case for *SLC45A3-ELK4,* found to be expressed in both normal prostate tissue and prostate cancer, with high levels of expression in a subset of prostate cancer samples. Only some prostate cancers expressing these fusion transcripts harbor chromosomal rearrangements at the corresponding genomic loci [20, 21]. The first recurrent fusion gene identified in CRC, *VTI1A-TCF7L2*, was originally detected in three of 97 CRCs, and found to be caused by a genomic deletion in the NCI-H508 CRC cell line [22]. However, when probed for with sensitive PCR, expression of *VTI1A-TCF7L2* fusion transcript was seen in a higher frequency of both normal and malignant tissue, probably as a result of read-through splicing [23]. The presence of splicing-generated fusion transcripts in normal cells and corresponding chromosome rearrangements followed by overexpression in cancer has been proposed to be a linked mechanism [24]. Intragenic deviating expression patterns can be caused by different promoter strengths of two fusion partner genes, usage of alternative core promoters or differential splicing. Exon-level microarrays, with probe sets in each annotated exon, as well as RNA sequencing technologies, enable investigation of complicated structural transcription events in cancer.

In this study, we have used exon-level expression data from a series of CRC as a screening tool to identify genes with differential internal expression, which can be indicative of their involvement as partner genes in fusion transcripts or being transcribed from different promoters. The transcript structures of nominated candidate genes were investigated by a combination of traditional rapid amplification of cDNA ends (RACE) and high-throughput RNA sequencing. This combination of methods facilitated the identification of fusion transcripts and transcript variants overrepresented in CRC.

## RESULTS

**Figure 1:**
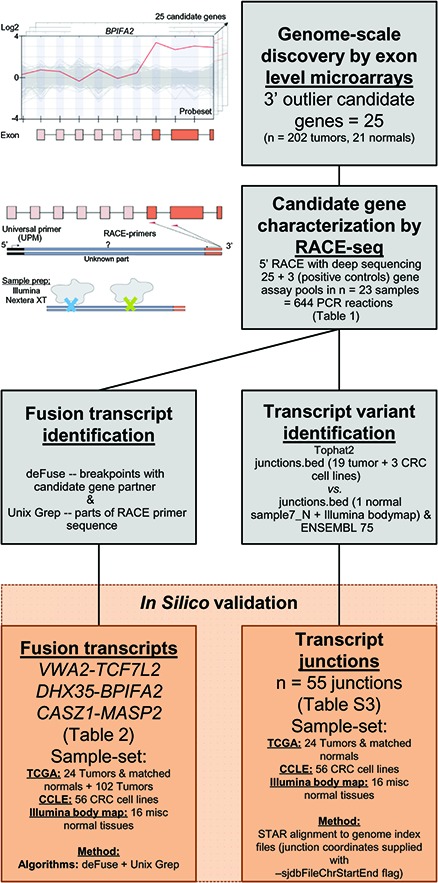
Pipeline to identify and characterize novel RNA variants in CRC Analysis of genome-scale exon level microarray data revealed 25 candidate genes with overexpression of their 3′ parts, here exemplified with the *BPIFA2* gene. The candidate genes were characterized with RACE-seq, a combination of 5′ RACE and deep sequencing. For the 25 candidate genes and also 3 positive control genes, nested RACE-primers were designed downstream of the suspected breakpoints (orange arrows). The resulting pools of RACE fragments (28 assays per sample) were prepared for sequencing with the Nextera XT protocol (Illumina), using tagmentation to simultaneously fragment and tag RACE-amplicons with adapters for sequencing. The fusion transcripts *VWA2-TCF7L2, DHX35-BPIFA2* and *CASZ1-MASP2*, and also 55 transcript junctions were identified by two separate computational approaches from the RACE-seq data. These were probed for in external datasets from TCGA, CCLE and the Illumina body map.

From exon-level genome-wide microarray data of 202 CRCs, we selected 25 genes with increased expression of their 3′ parts in at least one cancer sample (Table 1). These abnormal expression profiles typically reflect that the gene is transcribed by an alternative and stronger promoter, either within the gene itself, or from a separate gene. Twenty-four of the top-scoring genes were targeted by more than one probe set at both sides of the respective breakpoints, while one candidate (*FABP7*) obtained a very high expression break score (EBS) and was included even if it was targeted only by a single probe set 5′ to the putative breakpoint. Exon-level expression profiles for all candidate genes can be found in supplementary Figure S1. One candidate gene (*S100A2*) has previously been nominated from RNA sequencing data as a downstream partner of three fusion transcripts in the CRC cell line RKO, with *ZNF833*, *RP1–28O10.1*, and *AMPD3* as 5′ fusion partners [25]. An overview of the pipeline used to identify and characterize novel RNA variants in CRC is provided in Figure 1.

**Table 1:** Top 25 genes with elevated 3′ expression in individual CRCs

Gene symbol	Ensembl ID	Chromosome	Strand	Deviating sample	Expression Break score (EBS)	Type
*ACY3*	ENSG00000132744	11	−1	Sample12_T	2.60	5′ RACE candidate
*ASPRV1*	ENSG00000244617	2	−1	Sample5_T	4.00	5′ RACE candidate
*BAAT*	ENSG00000136881	9	−1	Sample13_T	3.67	5′ RACE candidate
*BPIFA2*	ENSG00000131050	20	1	Sample16_T	2.65	5′ RACE candidate
*CA6*	ENSG00000131686	1	1	Sample9_T	4.16	5′ RACE candidate
*COLGALT2*	ENSG00000198756	1	−1	Sample19_T	4.67	5′ RACE candidate
*FABP7*	ENSG00000164434	6	1	Sample10_T	5.67	5′ RACE candidate
*FGF12*	ENSG00000114279	3	−1	Sample12_T	3.37	5′ RACE candidate
*GUCY1A2*	ENSG00000152402	11	−1	Sample1_T	3.68	5′ RACE candidate
*HOXC12*	ENSG00000123407	12	1	Sample15_T	4.61	5′ RACE candidate
*IL11*	ENSG00000095752	19	−1	Sample20_T	3.47	5′ RACE candidate
*INA*	ENSG00000148798	10	1	Sample14_T	5.73	5′ RACE candidate
*KLK7*	ENSG00000169035	19	−1	Sample2_T	3.91	5′ RACE candidate
*KRT24*	ENSG00000167916	17	−1	Sample5_T	3.45	5′ RACE candidate
*LY6D*	ENSG00000167656	8	−1	Sample5_T	3.15	5′ RACE candidate
*LYPD3*	ENSG00000124466	19	−1	Sample5_T	5.06	5′ RACE candidate
*MASP2*	ENSG00000009724	1	−1	Sample4_T	3.25	5′ RACE candidate
*MOGAT1*	ENSG00000124003	2	1	Sample6_T	2.29	5′ RACE candidate
*MUC15*	ENSG00000169550	11	−1	Sample5_T	3.81	5′ RACE candidate
*NINJ2*	ENSG00000171840	12	−1	Sample8_T	3.40	5′ RACE candidate
*SOHLH2*	ENSG00000120669	13	−1	Sample3_T	2.75	5′ RACE candidate
*S100A2*	ENSG00000196754	1	−1	Sample5_T	5.85	5′ RACE candidate
*SLC22A2*	ENSG00000112499	6	−1	Sample17_T	4.33	5′ RACE candidate
*SLC38A11*	ENSG00000169507	2	−1	Sample19_T	4.54	5′ RACE candidate
*SNAP25*	ENSG00000132639	20	1	Sample7_T	3.08	5′ RACE candidate
*RP11–57H14.3*	ENSG00000225292	10	1	HCT116	NA	Positive control
*TCF7L2*	ENSG00000148737	10	1	NCI-H508	NA	Positive control
*VNN1*	ENSG00000112299	6	−1	HT29	NA	Positive control

The top 25 genes with elevated 3′ expression, identified by analysis of exon microarray data, were selected for follow-up transcript characterization by 5′ RACE and sequencing. In addition to the 25 top-scoring genes, three genes with previously known transcriptional changes were included as positive controls.

### Novel fusion transcripts in CRC

The 25 nominated candidate genes and three positive control genes were analyzed by 5′ RACE in all samples with highest EBS (*n* = 23; *i.e*. a total of 644 RACE reactions). The RACE-products from all gene assays for each sample were then pooled and sequenced, generating 15.5 million pairs of sequencing reads. After demultiplexing, trimming away 5′ RACE adapter sequences, and quality assurance, 12.6 million pairs remained (Table S1). Trimming 5′ RACE adapter sequences increased the number of paired-end reads aligned by up to 79% for each individual sample (Table S1).

**Figure 2:**
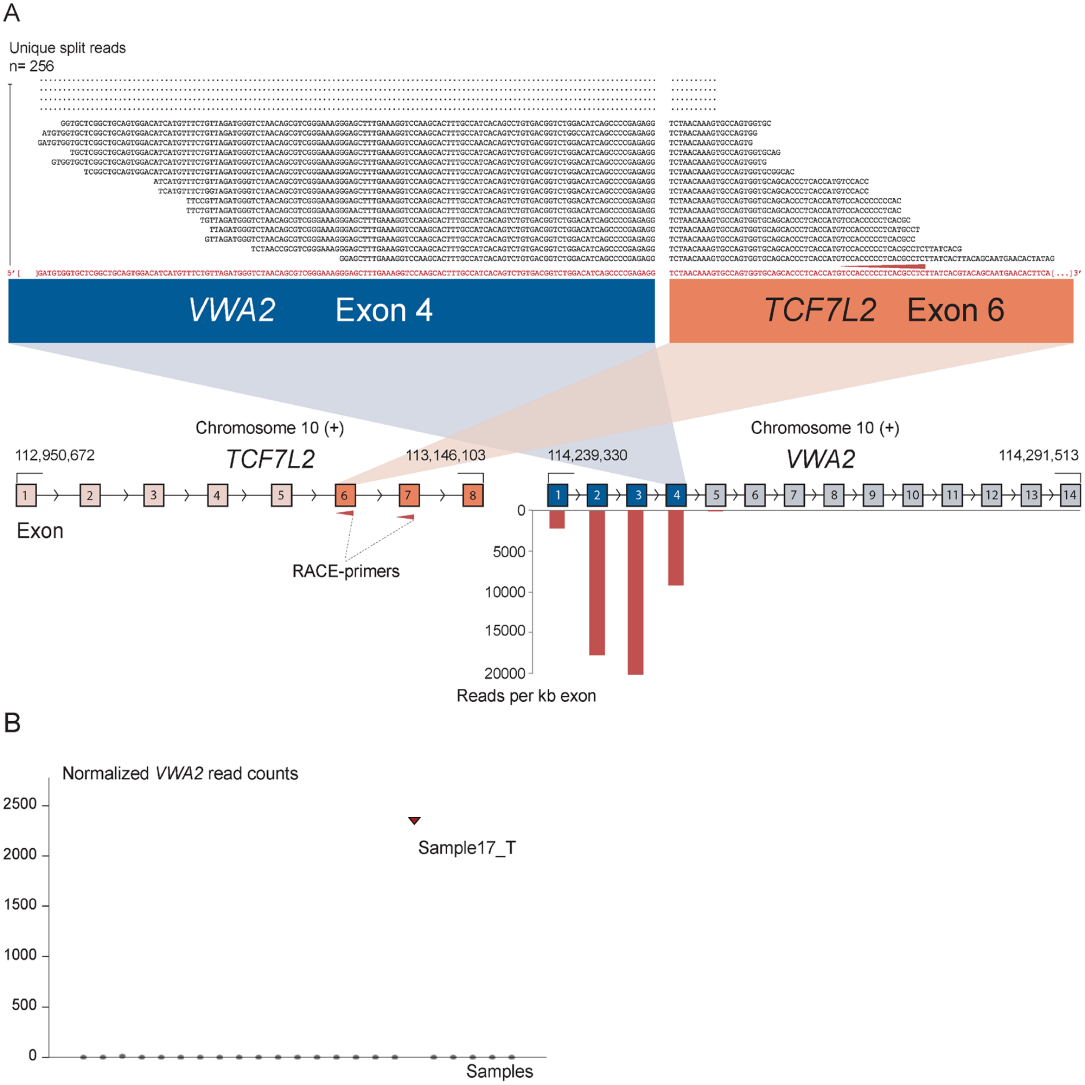
The novel fusion transcript *VWA2-TCF7L2* **A.** As indicated by red arrows, a RACE assay with first and second round primers targeting exon 7 and 6 of *TCF7L2* respectively was included in the sequencing setup as a positive control. In addition to enabling identification of previously known fusions involving *TCF7L2*, a new fusion was discovered that connects exon 4 of *VWA2* to exon 6 of *TCF7L2.* The exon numbers refer to transcript structures ENST00000392982 for *VWA2* and ENST00000369395 for *TCF7L2*. The 5′ partner gene, *VWA2,* is located 1.1 Mbp downstream of *TCF7L2*. The four exons upstream of the fusion breakpoint in *VWA2* show high read coverage, measured in reads per kilobase exon sequence (RPK). On the contrary, the exons after the breakpoint in *VWA2* were not picked up by the assay, indicating that the RACE assay targeting *TCF7L2* specifically amplified the upstream *VWA2* part in the tumor sample harboring the *VWA2-TCF7L2* fusion. **B.** Normalized read counts mapping to the *VWA2* gene for all samples included in the RACE-seq experimental set up. Only the sample with the identified *VWA2-TCF7L2* fusion transcript had sequencing reads covering the *VWA2* genes.

**Figure 3:**
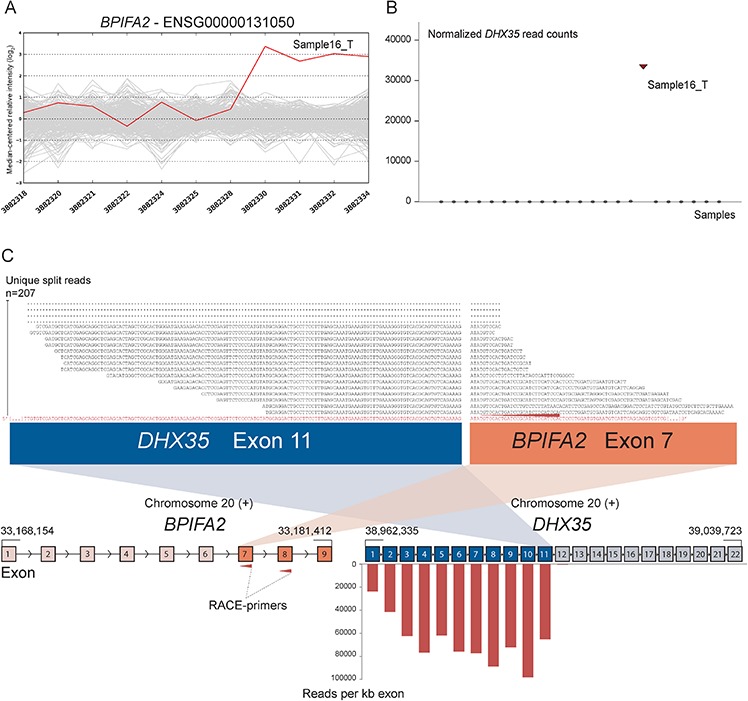
Novel fusion between *DHX35* and *BPIFA2* is in concordance with 3′ overexpression of *BPIFA2* **A.** The exon expression profile of *BPIFA2* shows that one CRC sample has a 3-fold increase in expression of the 3′ part of the gene compared to the median of the cohort. The last four probe sets showed increased intensity levels and target exon 7, 8 and 9 of *BPIFA2*. **B.** Normalized read counts mapping to *DHX35* were high only in sample16_T, the same sample which exhibits increased 3′ expression of *BPIFA2*. **C.** From RACE-seq we identified a fusion between exon 11 of *DHX35* and exon 7 of *BPIFA2*. Exons 1–11 of *DHX35* show high read coverage, measured in RPK. Exons 12–22, located downstream of the breakpoint in *DHX35*, were not covered by any reads, indicating that the RACE assay that targets *BPIFA2* specifically amplifies the upstream *DHX35* part of the fusion. By realigning sequencing reads from the sample in question to a fusion scaffold, we found that 207 unique split reads align across the fusion boundary.

**Figure 4:**
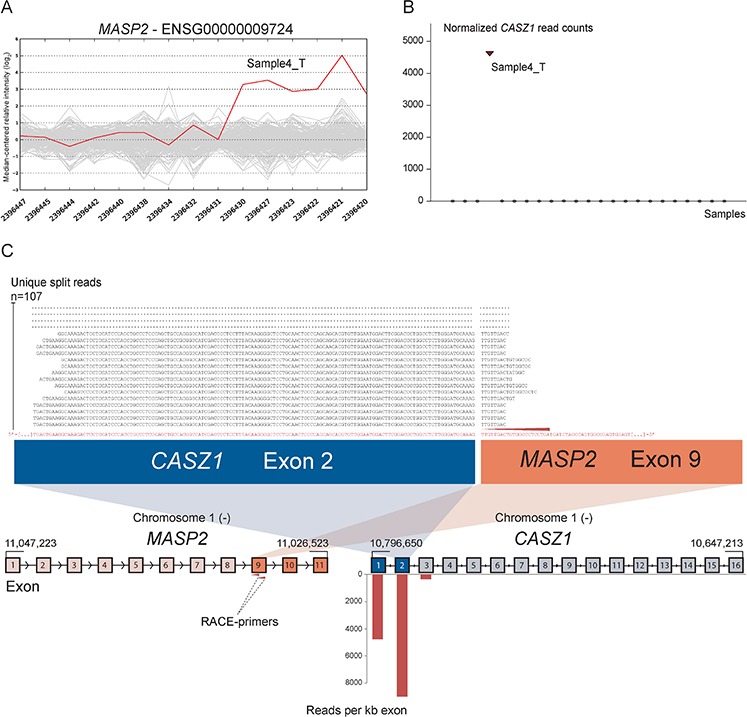
Novel fusion between *CASZ1* and *MASP2* is in concordance with 3′ overexpression of *MASP2* **A.** The exon expression profile of *MASP2* shows that one CRC sample has a 3- to 5-fold increase in expression of the 3′ part of the gene compared to the median of the cohort. The last six probe sets showed increased intensity and targeted exon 9, 10 and 11 of *MASP2.* **B.** Normalized read counts mapping to *CASZ1* were high only in sample 4_T, the same sample which exhibit increased 3′ expression of *MASP2*. **C.** From RACE-seq we identified that the gene *CASZ1* had high read coverage of its first two exons in the same sample that exhibited the deviating exon expression profile for *MASP2.* No fusions involving *MASP2* were nominated by deFuse, but when using the grep command for parts of the *MASP2* NGSP sequence (red arrow), we identified several reads containing a fusion boundary between *CASZ1* exon 2 and *MASP2* exon 9. Upon realigning sequencing reads, we found that 107 unique split reads aligns across the fusion boundary.

As positive controls for fusion gene detection by RACE-seq, we included *TCF7L2* and *RP11–57H14.3* as 5′ RACE targets in the NCI-H508 and HCT-116 cell lines, respectively. Both previously described fusions with these two genes as downstream partners were among the top nominated fusion breakpoints [22, 23] (Table 2). In addition to the two positive control fusions involving *TCF7L2*, another *TCF7L2* fusion with *VWA2* as a novel upstream fusion partner was identified in one tumor sample (Figure 2; Table 2). This sample also had a high number of reads covering the first four exons of *VWA2*. Several other genes, not directly targeted by 5′ RACE, showed similarly high coverage in individual tumor samples, indicating that they are indirectly amplified by the 5′ RACE assays (Figure S2). By evaluating sequence alignments for these genes, we identified two genes, *CASZ1* and *DHX35*, with high sequence coverage of the first two and 11 exons in individual samples, respectively. *DHX35* was identified as an upstream fusion partner of *BPIFA2* in the sample that had high sequence coverage of the first 11 exons of *DHX35* (Table 2; Figure 3). This sample was selected due to elevated expression of the 3′ part of the *BPIFA2* gene, which matched the identified fusion breakpoint. Similarly, the sample with elevated 3′ expression of the *MASP2* gene had high sample-specific coverage of the two first exons of *CASZ1* in the RACE-seq data (Figure 4). No fusion candidate with *CASZ1* as an upstream partner gene was nominated by the fusion detection software. However, the RACE assay for *MASP2* was designed to be in close proximity to the expression breakpoint observed in the exon microarray data. Thus, we used the Unix command-line utility grep to search for parts of the nested gene-specific primer (NGSP) sequence targeting *MASP2*. Using grep, several reads containing sequence from the *MASP2* NGSP mapping to both exon 9 of this gene and exon 2 of *CASZ1* were retrieved, indicating a fusion between these genes. After realigning raw reads to a fusion scaffold, we identified 107 split reads crossing the junction between *CASZ1* exon 2 (ENST00000344008) and *MASP2* exon 9 (ENST00000400897).

**Table 2:** Fusion transcripts detected by the RACE-seq approach

Gene A	Gene B	Break position A	Break position B	Split reads*	Spanning reads*	In Frame	Probability score^†^	Validated^§^	Sample
*VTI1A*	*TCF7L2*	Chr10: 114,220,341	Chr10: 114,900,943	1234	163	Yes	0.96	Previously reported	NCI-H508
*TCF7L2*	*RP11–57H14.3*	Chr10: 114,799,885	Chr10: 114,648,494	114	41	No	0.95	Previously reported	HCT-116
*VWA2*	*TCF7L2*	Chr10: 116,014,807	Chr10: 114,900,943	206	67	Yes	0.98	Yes	Sample 17_T
*DHX35*	*BPIFA2*	Chr20: 37,632,550	Chr20: 31,767,410	862	4	Yes	0.23	Yes	Sample 16_T
*CASZ1*	*MASP2*	Chr1: 10,820,757	Chr1: 11,090,938	107	NA	Yes	NA	Yes	Sample 4_T

Both previously known as well as novel fusion transcripts were detected by the RACE-seq approach and subsequent analysis of paired-end sequencing reads. Only the top-scoring nominated candidates are shown for each fusion pair. The known as well as the novel fusion transcripts *VWA2-TCF7L2* and *DHX35-BPIFA2* were nominated by the fusion detection software, whereas the novel *CASZ1-MASP2* was detected by the Unix utility grep and scaffold realignment.

* The number of split reads and spanning reads supporting the fusion junction, determined from the fusion detection software or scaffold realignment for the *CASZ1-MASP2* fusion (split reads contain the fusion boundary in the read itself, whereas spanning reads are paired ends that harbor the fusion boundary within the insert sequence).

† Probability score determined by the fusion detection software.

§ Two fusion genes involving *TCF7L2* have previously been reported and served as positive control fusion targets for the RACE-seq. The novel fusions were validated by Sanger sequencing.

### Fusion transcript validation

The three novel fusion transcripts were successfully validated using RT-PCR assays spanning the respective fusion boundaries followed by Sanger sequencing (Figure S3). All three fusion transcripts were generated by using intact splicing sites from their respective partner genes. The reading frames of the parental genes were retained for *VWA2-TCF7L2* and *DHX35-BPIFA2*. Both fusion transcripts potentially encode fusion proteins, as all four fusion partners have breakpoints within their coding sequences. The 5′ UTR of *CASZ1* is joined together with the 3′ part of the coding sequence of *MASP2*. Here, a start codon located 119 bp downstream of the fusion breakpoint encodes an open reading frame (ORF) that is in-frame with the reference *MASP2* ORF. As a consequence, the *CASZ1-MASP2* fusion transcript may encode an N-terminal truncated MASP2 protein under the control of the *CASZ1* promoter and 5′ UTR. None of the fusion transcripts were detected in external datasets from The Cancer Genome Atlas (TCGA) and the Cancer Cell Line Encyclopedia (CCLE), indicating that the fusions are private events.

### Novel transcript splice junctions in CRC

From the RACE-seq data we identified 147 novel splice junctions that had read coverage greater than 100 in at least one sample each (Table S2). From this list and by visual inspection of splicing junctions in the Integrated Genome Viewer (IGV), 55 junctions were selected that could potentially explain the deviating gene expression profiles in 20 of the genes (Table S3). In total, the RACE-seq data supported new transcript structures for 17 of the 23 targeted genes, not including the genes with validated fusion transcripts. Thirteen of the genes had sequence reads extending upstream from the annotated gene boundary, whereas 12 had sequence reads supporting new intragenic transcript structures including internal promoters and eight of the 23 genes had both.

We included the gene *VNN1* as a positive control for junction detection. We recently reported a novel transcript variant of this gene as being expressed in a large proportion of CRCs, including the HT29 cell line [8]. In addition to expression in HT29, 15 of the 20 primary carcinoma samples included in the RACE-seq expressed this transcript variant (Figure S4; Table S4), confirming previous results [8].

### New transcript variants of *S100A2* and *KLK7* are overrepresented in CRC

**Figure 5:**
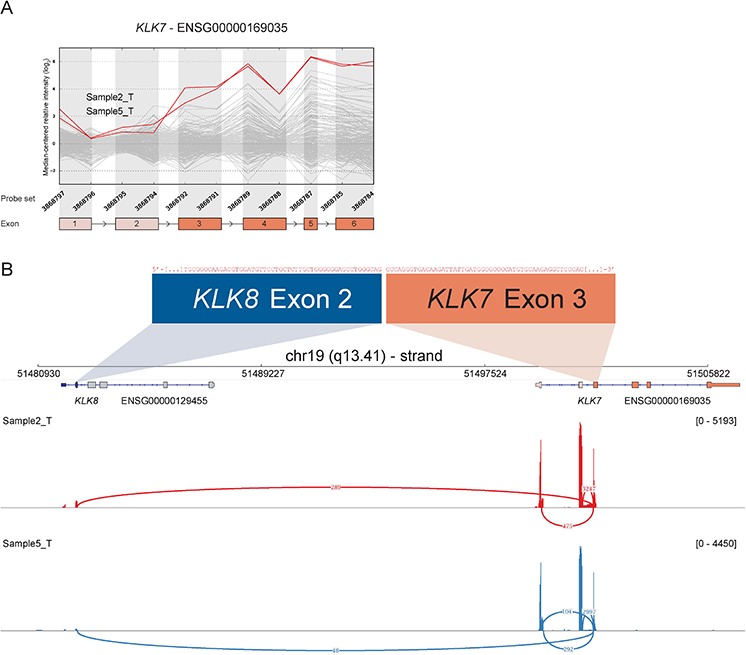
Read-through from upstream *KLK8* to *KLK7* in samples with deviating 3′ expression of *KLK7* **A.** The exon expression profile of *KLK7* shows that several samples have increased expression of probe sets targeting exon 3 to 6. The top two samples (2_T & 5_T) were selected for RACE-seq to identify underlying transcript structural changes. **B.** A sashimi plot from IGV shows the alignment of sequencing reads from the two nominated samples for *KLK7*. The height of the bars represents the number of aligned reads, while arcs represent junctions connected to exon 3 of *KLK7* and the coverage of these junctions, as determined by Tophat2 alignment and the sashimi plot package, are shown as numbers on the arcs.

RACE-seq data from the candidate genes *S100A2*, *KLK7*, *FGF12* and *BAAT* had reads supporting junctions of read-through fusion transcripts from upstream adjacent genes *S100A16*, *KLK8*, *MB21D2* and *MRPL50* respectively. The read-through *KLK8*-*KLK7* joins two members of the Kallikrein-related peptidase gene family that are 12 kb apart on chromosome 19. The exon microarray profile for *KLK7* showed increased expression of the 3′ part of the gene in several samples (Figure 5). The two samples with the highest EBS score were selected for RACE-seq. RACE-seq reads from both samples aligned to a junction between exon 2 of *KLK8* (ENST00000291726) and exon 3 of *KLK7* (ENST00000595820). The resulting read-through fusion transcript has an in-frame ORF, potentially encoding a fusion protein with coding sequences from both *KLK8* and *KLK7*. In total, eight of 19 CRC tumor samples and two of three CRC cell lines expressed this junction (Table 3 and Table S4). In the external datasets, six of 24 CRCs from the TCGA had at least one sequence read covering the *KLK8-KLK7* junction and 15 of 56 CRC cell lines had multiple sequence reads covering the junction. In contrast, the junction was not detected by sequence reads from any of the matching normal colonic mucosa samples, although a single read covered the junction in normal breast tissue in the Illumina human body map (Table S4).

**Table 3:** Validation frequencies of *KLK8-KLK7* and *S100A2* junctions

Gene	Chromosome	Pos 1	Pos 2	Distance	RACE-Seq*	TCGA_tumor*	TCGA_normal*	CCLE*	Body map
*KLK8-KLK7*	19	51485170	51504353	19183	10/22 (45%)	6/24 (25%)	0/24	15/56 (27%)	1/16^†^
*S100A2*	1	153536357	153537981	1624	16/22 (73%)	14/24 (58%)	0/24	33/56 (59%)	1/16^†^

The novel junctions covering the read-through of *KLK8* and *KLK7* and the alternative 3′ splice site of *S100A2* were discovered from the RACE-seq data. The junctions were validated by aligning all RACE-seq sequences and external data sets from the TCGA and the CCLE.

* The number of reads for the junctions to be considered positive were >10, >=2 and >= 1 for the RACE-seq, CCLE and TCGA tumor/normal data sets, respectively.

† From the Illumina Human body map data set, one read covering the *KLK8-KLK7* read-through was identified in normal breast tissue, and 12 reads were found to cover the *S100A2* alternative splice site in normal lung tissue.

**Figure 6:**
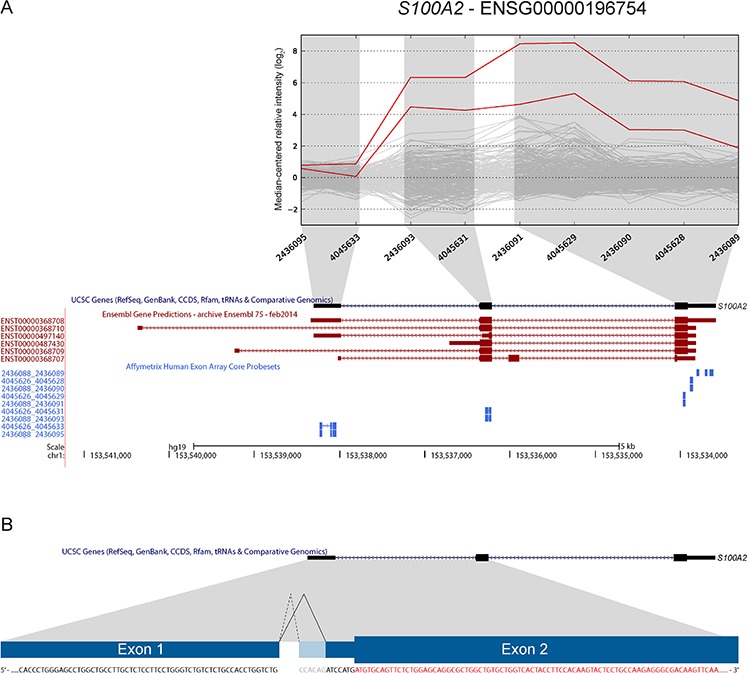
A novel 3′ splice-site in *S100A2* is found to be overrepresented in CRC **A.** Two samples had increased expression levels of probe sets targeting exons 2 and 3 of *S100A2* (UCSC annotation). Ensembl release 75 has annotated additional transcript variants (in red; ENST00000368710, ENST00000368709) and upstream 5′ UTR exons that are not targeted by exon microarray probe sets. **B.** We identified the use of a novel 3′ splice-site when splicing exon 1 to exon 2. The splice-site is located 6 bp downstream of the canonical splice-site. Use of this alternative splice-site was found to be overrepresented in CRC samples (see Table 3). The splice-site occurs in the 5′ UTR (coding sequence in red), and according to our analysis does not alter the annotated ORF.

*S100A2* was included based on increased expression of the 3′ part in two samples (Figure 6). Our approach to identify new junctions revealed alternative splicing between exon 1 and 2, with a new 3′ splice site only six bp downstream of the canonical splice site (ENST00000368708). Although this junction alone could not explain the deviating exon expression profiles, it was found to be overrepresented in CRC. In total, the splice site was covered by sequencing reads in 13 of 19 CRCs and in all three CRC cell lines (Table 3). Furthermore, 14 of 24 (58%) of the TCGA tumor samples had at least one read covering the junction, while 33 of 56 (59%) CRC cell lines had multiple reads covering the splice junction. Importantly, none of the matched normal samples from the TCGA had any reads covering the junction. In the Illumina human body map dataset, only one sample from normal lung tissue was found to have 12 reads covering the same splice junction.

## DISCUSSION

We have identified a set of novel transcript variants in CRC by a novel RACE-seq approach following an exon-level transcriptomics screen enabling simultaneous detection of variants from multiple genes and samples. Proof-of-concept was demonstrated by detection of known fusion transcripts involving *TCF7L2*, and alternative promoter usage in the *VNN1* gene.

Among the three novel private fusions transcripts, *VWA2* was detected as a new and previously unknown fusion partner of *TCF7L2*, strengthening the hypothesis that this WNT-effector transcription factor is involved in CRC. Interestingly, the *VWA2*-*TCF7L2* fusion transcript shares the same breakpoint of *TCF7L2* as the known *VTI1A*-*TCF7L2* fusion. Knock-down of *VTI1A*-*TCF7L2* in the NCI-H508 CRC cell line was previously shown to inhibit anchorage-independent growth [22]. The *DHX35* gene has previously been identified as a 5′ fusion partner to the *ITCH* gene in the SK-BR-3 breast cancer cell line [26], indicating that it may be a 5′ partner in multiple fusion genes. All three fusions were predicted to encode intact ORFs, implying a potential functional role.

In a similar approach to ours, the Encyclopedia of DNA elements (ENCODE) performed 5′ RACE combined with high-density resolution tiling microarrays to annotate transcript products from 399 known protein-coding loci. The results revealed that >80% of the tested genes had unannotated transcribed fragments both upstream and internal to previously known gene boundaries [27]. The ENCODE project has suggested that up to three-quarters of the human genome is capable of being transcribed, and that the current concept of a gene is in need of refinement [28]. Our data are in line with this, inasmuch as 17 of 23 candidate genes have reads supporting new transcript structures, and 13 have reads extending upstream of the currently annotated gene boundaries.

Among the identified transcript variants, we found a recurrent read-through fusion transcript between *KLK8* and *KLK7* and a novel 3′ splice site in *S100A2*. These were both found to be overrepresented in CRC tumor samples and cell lines when investigated in external RNA-seq datasets from TCGA, CCLE and the Illumina Human body map. *KLK7* encodes a serine protease of the kallikrein-related peptidases, and has previously been shown to be overexpressed in CRC. *KLK7* overexpression has also been found to be associated with increased cell proliferation *in vitro* and increased tumor growth in nude mice [29]. Furthermore, overexpression of *KLK7* mRNA and protein product have been related to poor clinical prognosis of CRC patients [30, 31], and the use of alternative promoters has been indicated in tissue or disease-specific *KLK* regulation [32]. The *KLK8-KLK7* read-through, with an intact ORF and overrepresentation in CRC, has biomarker potential and may have functional consequences for the disease which warrant further studies. The observed increase in expression of 3′ exons of *S100A2* is likely explained by the additional transcript variants annotated by Ensembl (Figure 6). However, the identification of a new 3′ splice site, detected in more than 50% of both primary CRC tumors and CRC cell lines but none of the matched normal samples makes it a potential cancer-specific splice variant. *S100A2* is a member of the S100 gene family, where S100 proteins are Ca^2+^ binding proteins with broad implications in cancer [33]. Moreover, *S100A2* expression has been proposed as a prognostic biomarker for tumor recurrence in CRC patients treated with adjuvant chemotherapy after surgery [34]. Although theoretically not altering the ORF and the subsequent translated protein, the alternative 3′ splice usage in *S100A2* identified here may have other regulatory or functional roles in CRC.

In terms of methodology, the idea of combining 5′ RACE with transcript variant characterization using paired-end sequencing shows potential for the identification of tissue- or disease-specific transcriptional structural changes. High-throughput sequencing characterization of RACE amplicons is highly time-efficient, more sensitive and technically feasible compared to traditional characterization of RACE fragments with cloning, plasmid isolation and Sanger sequencing. In our setup, we computationally nominated candidate genes with increased 3′ expression in CRC samples from exon microarray data to identify novel cancer-specific markers. However, the combination of 5′ RACE with paired-end sequencing also has other potential applications, such as testing for known fusion genes across tumor types or within different clinical cohorts. A future improvement would be to design RACE assays sufficiently downstream of suspected breakpoints to ensure sequencing reads on both ends, giving traditional fusion detection algorithms enough power to detect all fusions.

Using a novel high-throughput approach, we identified three previously unknown private fusion transcripts, and new transcript structures in 17 of the 23 other candidate genes. The novel transcripts included a recurrent read-through transcript between *KLK8* and *KLK7* and use of an alternative 3′ splice site in *S100A2* that are overrepresented in CRC. Both *KLK7* and *S100A2* have previously been implicated in CRC, making these transcript variants interesting as candidate markers in CRC.

## MATERIALS AND METHODS

### Patient samples and cell lines

A consecutive series of 202 primary colorectal carcinomas (stage I-IV) and normal colonic mucosa samples from 21 of the patients were included in the analysis. The CRC series was collected between 2005 and 2009 at Aker University Hospital, Oslo, Norway. Three CRC cell lines HT29, HCT116 and NCI-H508, were used in the study. They were obtained from American Type Culture Collection (Manassas, VA, USA), and were authenticated by in-lab STR analysis [35].

Research on the biomaterial, including with use of deep sequencing technology, was approved by the Regional Ethics Committee of South-Eastern Norway (2010/1805/REK south-east C).

### Exon level gene expression analysis

We previously performed genome-scale exon-level analyses of gene expression for 125 CRC and 21 normal mucosa samples [36–38] that have been deposited in the NCBI's Gene Expression Omnibus [GEO; accession numbers GSE24550, GSE29638, GSE42690]. Here an additional 77 CRCs from the same series are analyzed using the Affymetrix HuEx-1_0-st-v2 arrays. The samples were prepared and hybridized onto the arrays according to the manufacturer's instructions, as previously described [36]. From scanned images of the microarrays, cell intensity (CEL) files were generated by the Affymetrix GeneChip Command Console software (version 1.0). Using the Affymetrix Expression Console software (version 1.1), raw data was preprocessed by background correction of individual probes based on GC-content, inter-chip quantile normalization, and eventually summarized on the exon (core probe set) level by the robust multi-array average approach [39]. Raw and processed data are deposited in GEO under accession number GSE69182. For each probe set, the log2 expression signal of each sample relative to the median of that probe set across all samples was calculated. For the RACE analyses of candidate genes, we included 19 of the 202 CRCs with significant deviating exon-level expression profiles for at least one of the candidate genes each, and one normal colonic mucosa sample, as well as the three CRC cell lines (HT29, HCT116 and NCI-H508).

### Computational selection of fusion transcript candidates

An algorithm was developed to detect samples that possess abnormal expression profiles from the exon microarray data. The microarray data consists of intensities measured by a total number of 287,329 probe sets with an average of four probes per exon annotated by RefSeq [40] and full-length mRNA GenBank records [41]. To detect samples that diverge from the rest of the set, normalization by subtraction of the median signal intensity was applied for each individual probe set. Normalization was followed by division of the standard deviation at each probe set. Sample *i* is assigned a normalized relative expression value at probe set *j* that equals

$$s_{i,j}=\frac{p_{i,j}-\mu_{j}}{\sigma_{j}}$$

where *p_i,j_* is the log signal intensity, and *μ_j_* and σ_*j*_ are the median value and the standard deviation across all samples at probe set *j*, respectively. The EBS for sample *i* in combination with a particular gene *g* that is targeted by *k* probe sets equals the maximum magnitude of the difference of means from 5′ to 3′ along the gene:

$${\text{EBS}}_{ig}\;=\;\underset{j=1,\ldots ,k-1}{\text{max}}\left|\text{mean}\left(s_{i,j+1},\ldots ,s_{i,k}\right)-\text{ mean}\left(s_{i,1},\ldots ,s_{i,j}\right)\right|,$$

where *k* is the position of the putative breakpoint along the 5′ to 3′ axis of the gene. Large EBS_*ig*_ is therefore indicative of candidates where the expression level of sample *i* deviates from the rest of the set in either end of gene *g*. In most cases, expression of fusion transcripts is regulated by the promoter of the 5′ fusion partner [42]. Thus, the 3′ fusion partners often show increased signal intensities downstream of the breakpoint, due to the influence of a more active promoter. Only candidates with increased expression of the 3′ part, and thus being potential 3′ fusion partners, were nominated for further analysis. Genes with elevated EBS in any of the normal samples were discarded from further analyses. Top-scoring gene candidates were manually curated by inspecting the probe sets underlying the deviating exon expression profiles in the Annmap database [43]. Gene candidates with deviating exon expression profiles caused by probe sets mapping to several paralogous gene copies in the genome were filtered out, as well as profiles that were likely to be caused by alternative transcription of already annotated transcript variants. For robustness, the algorithm selected only fusion partner candidates where more than one probe set had different intensity levels.

### 5′ Rapid amplification of cDNA ends (RACE)

We designed nested RACE-PCR assays for each of the candidate genes and three genes used as positive controls (*TCF7L2*, *RP11–57H14.3*, and *VNN1*). Based on the exon-level expression profiles, a reverse gene-specific primer (GSP) and a reverse NGSP were designed against sequences downstream of the expected breakpoints. A forward internal control primer (ICP) was designed against sequences upstream of the same gene, to be used as a positive control and for primer optimization. To enable touchdown PCR, all primers were designed using primer 3 software [44] with theoretical T_m_ > 70°C, GC content 50–70% and length 23–28 nucleotides. Primer sequences for all gene assays are listed in Table S5. To obtain the full-length 5′ end sequence of the mRNA transcripts, 5′ RACE-ready cDNA was synthesized from 1 μg total RNA for each of the 24 samples using the SMARTer™ RACE cDNA Amplification kit according to protocol (Clontech, Mountain View, CA, USA). This technology incorporates the use of an oligo (dT) primer in combination with a SMARTer II A oligonucleotide adapter and the SMARTScribe Reverse Transcriptase, which in effect generates modified cDNAs containing 5′ adapter sequences from all transcribed mRNAs. Products were diluted in 100 μl milli-Q water. Synthesis of 5′ RACE-ready cDNA was confirmed by a control PCR assay detecting the housekeeping gene *GUSB*. Additionally, negative no-template controls were included in each set-up to control for contamination.

First-round RACE-PCR was performed for all candidate genes in a 10 μl reaction volume. 5′ RACE-ready cDNA from the 24 samples was diluted 4x before 2 μl of this dilution was used as input. In total, 672 first round RACE-PCR reactions were performed separately. Touchdown PCR was used, which started with 5 cycles of denaturation at 94°C for 30 seconds, annealing and extension at 72°C for 3 minutes, followed by 5 cycles of denaturation at 94°C for 30 seconds, annealing at 70°C for 30 seconds and extension at 72°C for 3 minutes, and finally, 25 cycles with denaturation at 94°C for 30 seconds, annealing at 68°C for 30 seconds and extension at 72°C for 3 minutes. The first-round PCR products were diluted 50x and 2.5 μl of each was used as template input into the nested RACE-PCR. The nested RACE-PCR reactions were carried out in a 25 μl reaction volume for each assay in each of the samples. To normalize the amounts of nested RACE products from each reaction we used a quantitative DNA binding approach (SequalPrep™ Normalization Plate Kit, 96-well; Applied Biosystems^®^ by Life Technologies, Carlsbad, CA, USA). We added 20 μl nested RACE products to each well in the normalization plates and continued according to the manufacturer's protocol. For each sample, equal volumes of normalized amplicons from the 28 different assays were pooled together. The pools were further quantified using the Qubit^®^ 2.0 Fluorometer and Qubit^®^ dsDNA HS Assay Kit. One sample was found to have insufficient amount of pooled amplicons, leaving 23 samples for sequencing. For each sample, 1 ng of the nested RACE product pools was used as input to the Nextera XT sample preparation protocol.

### Sample preparation for MiSeq sequencing

The Nextera XT sample preparation kit from Illumina (San Diego, CA, USA) was used. We performed tagmentation of the input nested RACE products, before performing 12 cycles amplification of the fragments, both according to the manufacturer's protocol. During amplification, dual indexes were added to enable multiplexing of the nested RACE product pools. The amplicon pools were further cleaned and size selected using 0.6x Ampure XP beads. A subset of cleaned libraries was quality controlled using Bioanalyzer HS DNA chips. After clean up, we normalized the pools using the provided library normalization beads. To pool the samples, 5 μl of each library was combined in an Eppendorf tube and 24 μl of the pool was mixed with 576 μl of hybridization buffer. The library pool was then loaded onto a thawed MiSeq reagent cartridge version v2 before 2 × 150 nucleotides sequencing in the MiSeq instrument.

### Identification of upstream fusion partners

After demultiplexing the paired-end reads, fastq files from the individual samples were subjected to quality control and inspection by using the FastQC software [45]. The sequencing reads were further trimmed to remove bad quality ends, and also to remove SMARTer II adapter oligos that were adhered during RACE cDNA synthesis. Trimming and filtering was done using the cutadapt software [46]. Trimmed reads from each sample were aligned to the Ensembl GRCh37 (iGenomes May 14 2014) reference using Tophat2 v.2.0.11 [47], Bowtie2 v.2.1.0 [48] and Samtools v.0.1.19 [49]. Normalized read counts were generated for the top 100 expressed genes using HTseq-count [50] and the R package DESeq2 [51].

For all samples, the gene fusion discovery software deFuse v.0.6.0 [52] was used to identify fusion transcripts that included one of the nominated candidate genes. In addition, normalized read counts were used to identify sample-specific expressed genes which were amplified using the 5′ RACE, but not among the original candidate genes. To determine which of these genes had high 5′ read coverage, visual inspection of aligned reads was done using the Integrative Genomics Viewer (IGV) [53, 54]. Genes identified in this manner were specifically searched for in the deFuse candidate lists. If not nominated by deFuse, the Unix command grep was used to find reads containing parts of the NGSP primer for the candidate gene in the specific sample. These reads were further aligned with BLAT [55] to identify if parts of the reads connected to the sample-specific expressed gene that was not targeted by the RACE gene-specific primers.

### *In silico* validation of fusion transcripts in external datasets

For *in silico* validation of fusion transcripts, raw RNA sequencing data (paired-end fastq files; 48 nucleotides read-length) from 126 CRCs and 24 matched normal colonic mucosa from TCGA (Table S6) was downloaded from The Cancer Genomics Hub (CGHub; https://browser.cghub.ucsc.edu/). As additional normal control samples, paired-end RNA sequencing data from the Illumina Human Body Map v2 dataset consisting of 16 non-malignant miscellaneous tissue types was downloaded (ArrayExpress accession ID E-MTAB-513 and European Nucleotide Archive study accession ID ERP000546; paired-end fastq files; 50 nucleotides read-length). Moreover, aligned BAM files were obtained for 56 colon adenocarcinoma cell lines from CCLE and converted back to paired end fastq files (101 nucleotides read-length; Table S7). The deFuse software was applied to detect genome-wide fusion candidates in the TCGA tumor and normal colonic mucosa samples, as well as in the CRC cell lines. Potential fusions were filtered against the Illumina Human Body Map v2 dataset. To specifically examine candidate fusion genes *DHX35-BPIFA2*, *CASZ1-MASP2*, *VWA2-TCF7L2*, and *VTI1A-TCF7L2*, the Unix command grep was used to search for scaffold sequences spanning 8, 10, 15, 20, and 24 bases at each side of the putative breakpoints.

### Identification of novel and cancer-specific transcript variants

Transcript splice junctions (*i.e*. possible cancer specific alternative splicing variants) were first identified from the junctions.bed files generated at the alignment step with Tophat2. The junctions.bed files from 19 CRC tumor samples and three CRC cell lines were merged. To keep only novel junctions, those previously annotated in Ensembl release 75 were removed. To select for cancer specificity, junctions that also were detected from the normal sample included in the RACE-seq as well as junctions from any of the 16 Illumina human body map tissues were filtered out. Finally, only junctions supported by a minimum of 100 split reads were kept for downstream analysis.

Transcript junctions that corresponded to intragenic differential exon-level expression for a candidate gene were selected from this list for *in silico* validation. Additional junctions were added for validation based on manual inspection of the corresponding sample's read alignment in IGV. Selected transcript junctions were used as input when generating genome index files (using the —sjdbFileChrStartEnd flag) from Ensembl release 75 with the STAR aligner v.2.4.0d [56]. Fastq files from the downloaded validation series were aligned to the generated genome index. In total, paired-end data from 24 matched tumor and normal pairs from the TCGA colon adenocarcinoma series, 56 colon adenocarcinoma cell lines from the CCLE and 16 miscellaneous tissues from the Illumina human body map were aligned to determine the presence of the selected transcript junctions.

### Availability of supporting data

The data sets supporting the results of this article are available in the NCBI's Gene Expression Omnibus repository, GSE24550 (http://www.ncbi.nlm.nih.gov/geo/query/acc.cgi?acc=GSE24550), GSE29638 (http://www.ncbi.nlm.nih.gov/geo/query/acc.cgi?acc=GSE29638), GSE42690 (http://www.ncbi.nlm.nih.gov/geo/query/acc.cgi?acc=GSE42690), GSE69182 (http://www.ncbi.nlm.nih.gov/geo/query/acc.cgi?token=qxobkqiyhvefbkn&acc=GSE69182).

## SUPPLEMENTARY DATA





## References

[1] Torre LA, Bray F, Siegel RL, Ferlay J, Lortet-Tieulent J, Jemal A (2015). Global cancer statistics, 2012. CA Cancer J Clin.

[2] Sotiriou C, Pusztai L (2009). Gene-Expression Signatures in Breast Cancer. N Engl J Med.

[3] Alizadeh AA, Eisen MB, Davis RE, Ma C, Lossos IS, Rosenwald A, Boldrick JC, Sabet H, Tran T, Yu X, Powell JI, Yang L, Marti GE (2000). Distinct types of diffuse large B-cell lymphoma identified by gene expression profiling. Nature.

[4] Sveen A, Nesbakken A, Ågesen TH, Guren MG, Tveit KM, Skotheim RI, Lothe RA (2013). Anticipating the Clinical Use of Prognostic Gene Expression-Based Tests for Colon Cancer Stage II and III: Is Godot Finally Arriving?. Clin Cancer Res.

[5] Venables JP (2004). Aberrant and Alternative Splicing in Cancer. Cancer Res.

[6] Skotheim RI, Nees M (2007). Alternative splicing in cancer: noise, functional, or systematic?. Int J Biochem Cell Biol.

[7] Kalvala A, Gao L, Aguila B, Reese T, Otterson GA, Villalona-Calero MA, Duan W (2015). Overexpression of Rad51C splice variants in colorectal tumors. Oncotarget.

[8] Løvf M, Nome T, Bruun J, Eknæs M, Bakken AC, Mpindi JP, Kilpinen S, Rognum TO, Nesbakken A, Kallioniemi O, Lothe RA, Skotheim RI (2014). A novel transcript, VNN1-AB, as a biomarker for colorectal cancer. Int J Cancer.

[9] Thorsen K, Mansilla F, Schepeler T, Oster B, Rasmussen MH, Dyrskjot L, Karni R, Akerman M, Krainer AR, Laurberg S, Andersen CL, Orntoft TF (2010). Alternative splicing of SLC39A14 in colorectal cancer is regulated by the Wnt pathway. Mol Cell Proteomics.

[10] Sveen A, Bakken AC, Ågesen TH, Lind GE, Nesbakken A, Nordgård O, Brackmann S, Rognum TO, Lothe RA, Skotheim RI (2012). The exon-level biomarker SLC39A14 has organ-confined cancer-specificity in colorectal cancer. Int J Cancer.

[11] Mitelman F, Johansson B, Mertens F (2004). Fusion genes and rearranged genes as a linear function of chromosome aberrations in cancer. Nat Genet.

[12] Mitelman F, Johansson B, Mertens F (2007). The impact of translocations and gene fusions on cancer causation. Nat Rev Cancer.

[13] Tomlins SA, Rhodes DR, Perner S, Dhanasekaran SM, Mehra R, Sun XW, Varambally S, Cao X, Tchinda J, Kuefer R, Lee C, Montie JE, Shah RB (2005). Recurrent fusion of TMPRSS2 and ETS transcription factor genes in prostate cancer. Science.

[14] Djebali S, Lagarde J, Kapranov P, Lacroix V, Borel C, Mudge JM, Howald C, Foissac S, Ucla C, Chrast J, Ribeca P, Martin D, Murray RR (2012). Evidence for Transcript Networks Composed of Chimeric RNAs in Human Cells. PLoS ONE.

[15] Frenkel-Morgenstern M, Lacroix V, Ezkurdia I, Levin Y, Gabashvili A, Prilusky J, Del PA, Tress ML, Johnson R, Guigo R, Valencia A (2012). Chimeras taking shape: Potential functions of proteins encoded by chimeric RNA transcripts. Genome Res.

[16] Plebani R, Oliver GR, Trerotola M, Guerra E, Cantanelli P, Apicella L, Emerson A, Albiero A, Harkin PD, Kennedy RD, Alberti S (2012). Long-range transcriptome sequencing reveals cancer cell growth regulatory chimeric mRNA. Neoplasia.

[17] Zhang Y, Gong M, Yuan H, Park HG, Frierson HF, Li H (2012). Chimeric Transcript Generated by cis-Splicing of Adjacent Genes Regulates Prostate Cancer Cell Proliferation. Cancer Discov.

[18] Yun SM, Yoon K, Lee S, Kim E, Kong S-H, Choe J, Kang JM, Han T-S, Kim P, Choi Y, Jho S, Yoo H, Bhak J (2014). PPP1R1B-STARD3 chimeric fusion transcript in human gastric cancer promotes tumorigenesis through activation of PI3K/AKT signaling. Oncogene.

[19] Frenkel-Morgenstern M, Gorohovski A, Vucenovic D, Maestre L, Valencia A (2014). ChiTaRS 2.1-an improved database of the chimeric transcripts and RNA-seq data with novel sense-antisense chimeric RNA transcripts. Nucleic Acids Res.

[20] Rickman DS, Pflueger D, Moss B, VanDoren VE, Chen CX, de la Taille A, Kuefer R, Tewari AK, Setlur SR, Demichelis F, Rubin MA (2009). SLC45A3-ELK4 is a novel and frequent erythroblast transformation-specific fusion transcript in prostate cancer. Cancer Res.

[21] Maher CA, Kumar-Sinha C, Cao X, Kalyana-Sundaram S, Han B, Jing X, Sam L, Barrette T, Palanisamy N, Chinnaiyan AM (2009). Transcriptome sequencing to detect gene fusions in cancer. Nature.

[22] Bass AJ, Lawrence MS, Brace LE, Ramos AH, Drier Y, Cibulskis K, Sougnez C, Voet D, Saksena G, Sivachenko A, Jing R, Parkin M, Pugh T (2011). Genomic sequencing of colorectal adenocarcinomas identifies a recurrent VTI1A-TCF7L2 fusion. Nat Genet.

[23] Nome T, Hoff AM, Bakken AC, Rognum TO, Nesbakken A, Skotheim RI (2014). High Frequency of Fusion Transcripts Involving TCF7L2 in Colorectal Cancer: Novel Fusion Partner and Splice Variants. PLoS One.

[24] Li H, Wang J, Ma X, Sklar J (2009). Gene fusions and RNA trans-splicing in normal and neoplastic human cells. Cell Cycle.

[25] Nome T, Thomassen GO, Bruun J, Ahlquist T, Bakken AC, Hoff AM, Rognum T, Nesbakken A, Lorenz S, Sun J, Barros-Silva JD, Lind GE, Myklebost O (2013). Common fusion transcripts identified in colorectal cancer cell lines by high-throughput RNA sequencing. Transl Oncol.

[26] Edgren H, Murumagi A, Kangaspeska S, Nicorici D, Hongisto V, Kleivi K, Rye IH, Nyberg S, Wolf M, Børresen-Dale A-L, Kallioniemi O (2011). Identification of fusion genes in breast cancer by paired-end RNA-sequencing. Genome Biol.

[27] Denoeud F, Kapranov P, Ucla C, Frankish A, Castelo R, Drenkow J, Lagarde J, Alioto T, Manzano C, Chrast J, Dike S, Wyss C, Henrichsen CN (2007). Prominent use of distal 5′ transcription start sites and discovery of a large number of additional exons in ENCODE regions. Genome Res.

[28] Djebali S, Davis CA, Merkel A, Dobin A, Lassmann T, Mortazavi A, Tanzer A, Lagarde J, Lin W, Schlesinger F, Xue C, Marinov GK, Khatun J (2012). Landscape of transcription in human cells. Nature.

[29] Walker F, Nicole P, Jallane A, Soosaipillai A, Mosbach V, Oikonomopoulou K, Diamandis EP, Magdolen V, Darmoul D (2014). Kallikrein-related peptidase 7 (KLK7) is a proliferative factor that is aberrantly expressed in human colon cancer. Biol Chem.

[30] Talieri M, Mathioudaki K, Prezas P, Alexopoulou DK, Diamandis EP, Xynopoulos D, Ardavanis A, Arnogiannaki N, Scorilas A (2009). Clinical significance of kallikrein-related peptidase 7 (KLK7) in colorectal cancer. Thromb Haemost.

[31] Talieri M, Li L, Zheng Y, Alexopoulou DK, Soosaipillai A, Scorilas A, Xynopoulos D, Diamandis EP (2009). The use of kallikrein-related peptidases as adjuvant prognostic markers in colorectal cancer. Br J Cancer.

[32] Christophi GP, Isackson PJ, Blaber S, Blaber M, Rodriguez M, Scarisbrick IA (2004). Distinct promoters regulate tissue-specific and differential expression of kallikrein 6 in CNS demyelinating disease. J Neurochem.

[33] Salama I, Malone PS, Mihaimeed F, Jones JL (2008). A review of the S100 proteins in cancer. Eur J Surg Oncol.

[34] Giráldez MD, Lozano JJ, Cuatrecasas M, Alonso-Espinaco V, Maurel J, Mármol M, Hörndler C, Ortego J, Alonso V, Escudero P, Ramírez G, Petry C, Lasalvia L (2013). Gene-expression signature of tumor recurrence in patients with stage II and III colon cancer treated with 5′fluoruracil-based adjuvant chemotherapy. Int J Cancer.

[35] Ahmed D, Eide PW, Eilertsen IA, Danielsen SA, Eknæs M, Hektoen M, Lind GE, Lothe RA (2013). Epigenetic and genetic features of 24 colon cancer cell lines. Oncogenesis.

[36] Sveen A, Ågesen TH, Nesbakken A, Rognum TO, Lothe RA, Skotheim RI (2011). Transcriptome instability in colorectal cancer identified by exon microarray analyses: Associations with splicing factor expression levels and patient survival. Genome Med.

[37] Agesen TH, Sveen A, Merok MA, Lind GE, Nesbakken A, Skotheim RI, Lothe RA (2012). ColoGuideEx: a robust gene classifier specific for stage II colorectal cancer prognosis. Gut.

[38] Sveen A, Johannessen B, Teixeira MR, Lothe RA, Skotheim RI (2014). Transcriptome instability as a molecular pan-cancer characteristic of carcinomas. BMC Genomics.

[39] Irizarry RA, Hobbs B, Collin F, Beazer-Barclay YD, Antonellis KJ, Scherf U, Speed TP (2003). Exploration, normalization, and summaries of high density oligonucleotide array probe level data. Biostatistics.

[40] Pruitt KD, Brown GR, Hiatt SM, Thibaud-Nissen F, Astashyn A, Ermolaeva O, Farrell CM, Hart J, Landrum MJ, McGarvey KM, Murphy MR, O'Leary NA, Pujar S (2014). RefSeq: an update on mammalian reference sequences. Nucleic Acids Res.

[41] Benson DA, Karsch-Mizrachi I, Lipman DJ, Ostell J, Wheeler DL (2006). GenBank. Nucleic Acids Res.

[42] Aman P (1999). Fusion genes in solid tumors. Semin Cancer Biol.

[43] Yates T, Okoniewski MJ, Miller CJ (2008). X:Map: annotation and visualization of genome structure for Affymetrix exon array analysis. Nucleic Acids Res.

[44] Rozen S, Skaletsky H (2000). Primer3 on the WWW for general users and for biologist programmers. Methods MolBiol.

[45] Andrews S FastQC a quality-control tool for high-throughput sequence data [Internet]. http://www.bioinformatics.babraham.ac.uk/projects/fastqc/.

[46] Martin M (2011). Cutadapt removes adapter sequences from high-throughput sequencing reads. EMBnet J.

[47] Trapnell C, Pachter L, Salzberg SL (2009). TopHat: discovering splice junctions with RNA-Seq. Bioinformatics.

[48] Langmead B, Trapnell C, Pop M, Salzberg SL (2009). Ultrafast and memory-efficient alignment of short DNA sequences to the human genome. Genome Biol.

[49] Li H, Handsaker B, Wysoker A, Fennell T, Ruan J, Homer N, Marth G, Abecasis G, Durbin R (2009). The Sequence Alignment/Map format and SAMtools. Bioinformatics.

[50] Anders S, Pyl PT, Huber W (2015). HTSeq—a Python framework to work with high-throughput sequencing data. Bioinformatics.

[51] Love MI, Huber W, Anders S (2014). Moderated estimation of fold change and dispersion for RNA-seq data with DESeq2. Genome Biol.

[52] McPherson A, Hormozdiari F, Zayed A, Giuliany R, Ha G, Sun MGF, Griffith M, Heravi Moussavi A, Senz J, Melnyk N, Pacheco M, Marra MA, Hirst M (2011). deFuse: An Algorithm for Gene Fusion Discovery in Tumor RNA-Seq Data. PLoS Comput Biol.

[53] Robinson JT, Thorvaldsdóttir H, Winckler W, Guttman M, Lander ES, Getz G, Mesirov JP (2011). Integrative genomics viewer. Nat Biotechnol.

[54] Thorvaldsdóttir H, Robinson JT, Mesirov JP (2013). Integrative Genomics Viewer (IGV): high-performance genomics data visualization and exploration. Brief Bioinform.

[55] Kent WJ (2002). BLAT-The BLAST-Like Alignment Tool. Genome Res.

[56] Dobin A, Davis CA, Schlesinger F, Drenkow J, Zaleski C, Jha S, Batut P, Chaisson M, Gingeras TR (2013). STAR: ultrafast universal RNA-seq aligner. Bioinformatics.

