# Supplementary material for: Novel RNA variants in colorectal cancers
Source: Oncotarget. 2015 Oct 12;6(34):36587–602. doi: 10.18632/oncotarget.5500 (PMC4742197; doi:10.18632/oncotarget.5500)
Supplement: Supplementary file 1 [file oncotarget-06-36587-s001.pdf]

SUPPLEMENTARY DATA

Supporting tables

Supporting tables are available in additional file 2, a Microsoft Excel Workbook with seven worksheets, one for each supplementary Table. All tables with genomic coordinates refer to hg19. Table S1: Sequence reads produced by high-throughput sequencing of RACE products, and alignment numbers before and after trimming. Table S2: Novel transcript splice junctions covered by a minimum of 100 reads from the RACE-seq data. Table S3: Transcript splice junctions used as input to the STAR aligner. Table S4: Number of aligned reads to input junctions, as determined by the STAR aligner. Table S5: Primers used in the RACE amplification of target genes. Table S6: RNA-seq data from The Cancer Genome Atlas, used for validation of fusion transcripts and splice junctions. Table S7: RNA-seq data from The Cancer Cell Line Encyclopedia used for validation of fusion transcripts and splice junctions.

Figure S1A

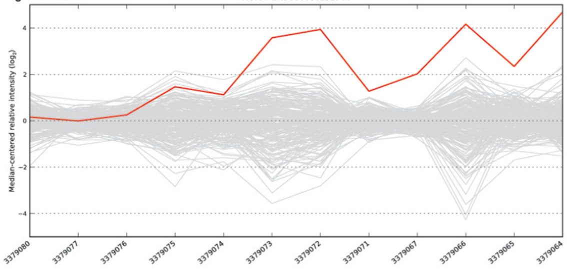

Figure S1B

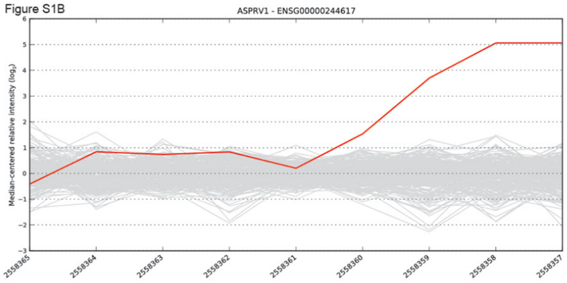

Figure S1C

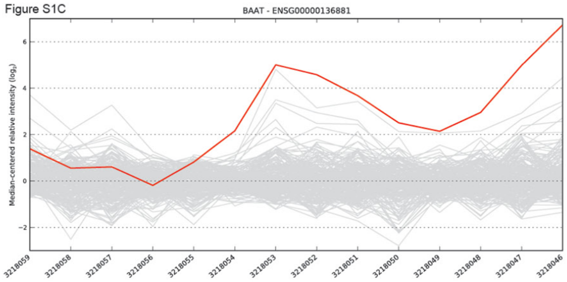

Figure S1D

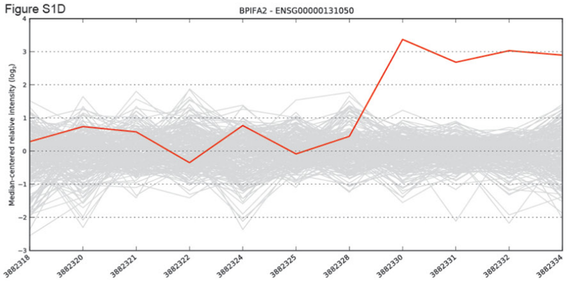

Figure S1E

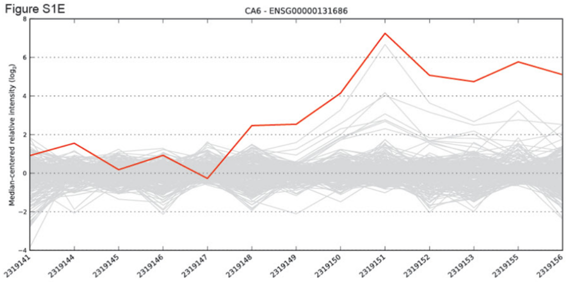

Figure S1F

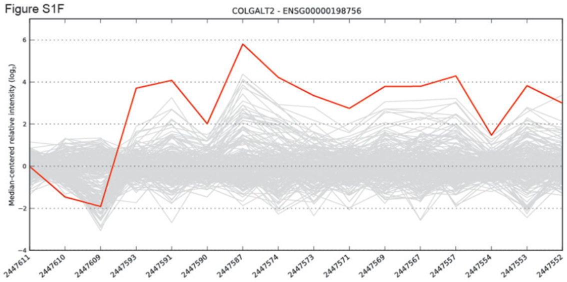

Figure S1G

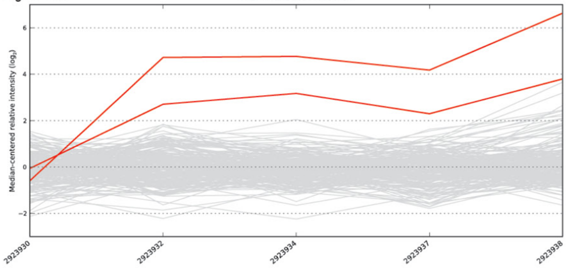

Figure S1H

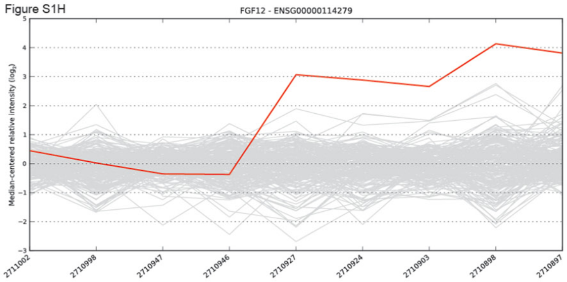

Figure S1I

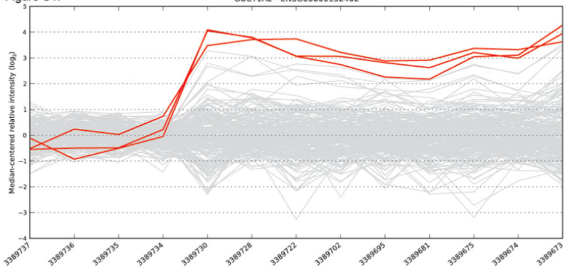

Figure S1J

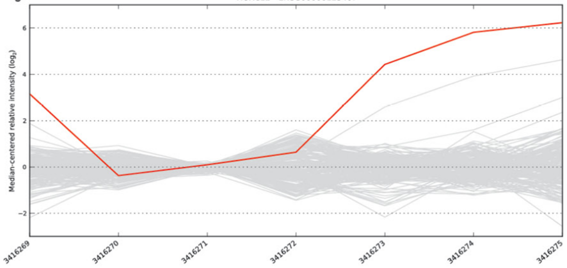

(*Continued*)

Figure S1K

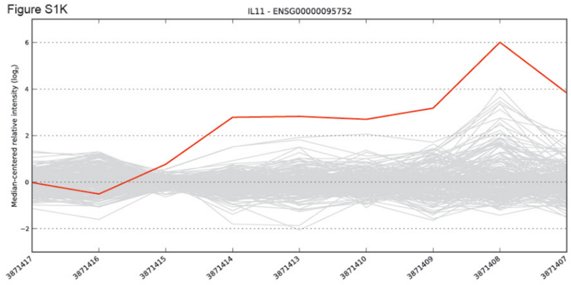

Figure S1L

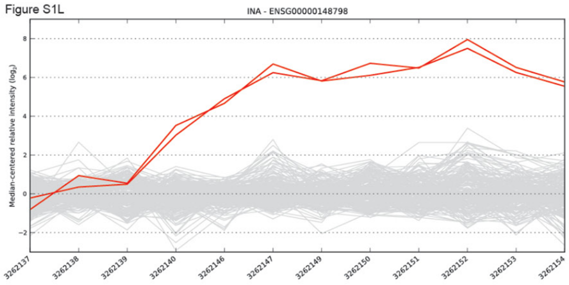

Figure S1M

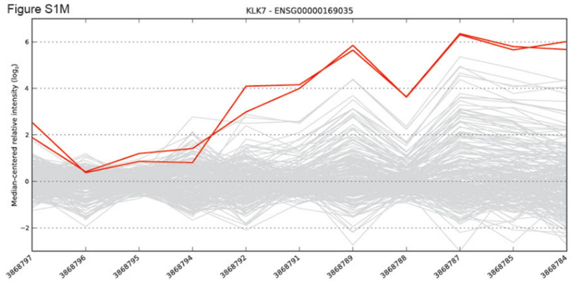

Figure S1N

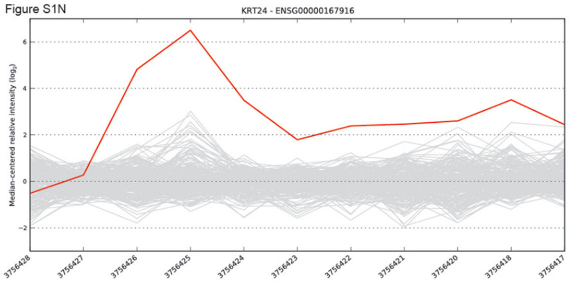

Figure S1O

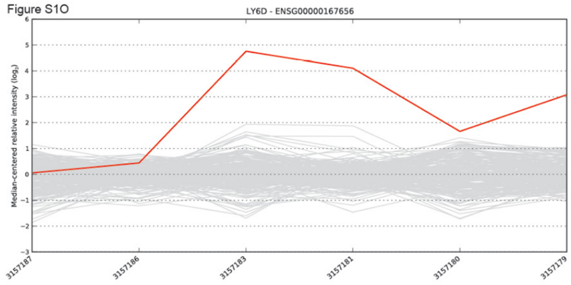

Figure S1P

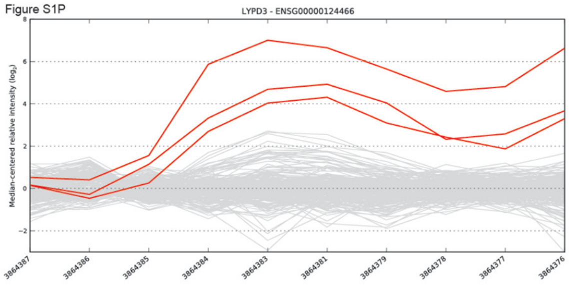

Figure S1Q

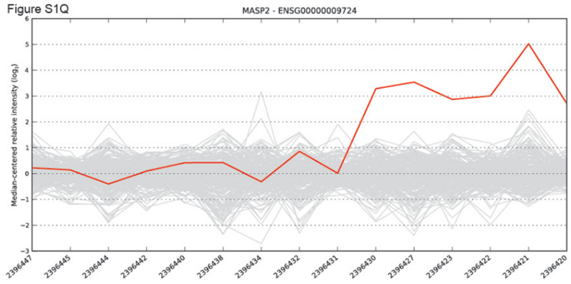

Figure S1R

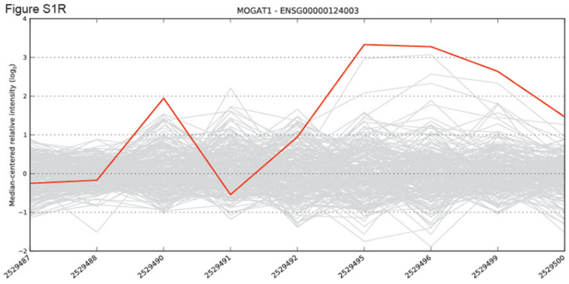

Figure S1S

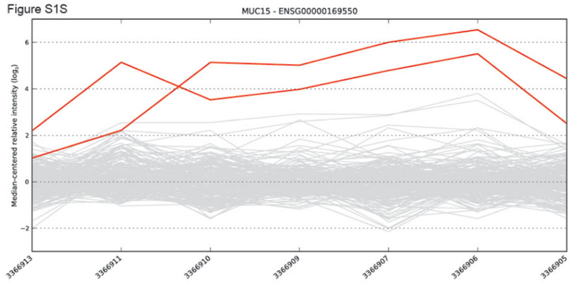

Figure S1T

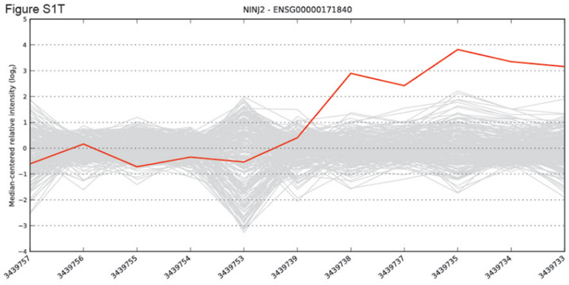

(*Continued*)

Figure S1U

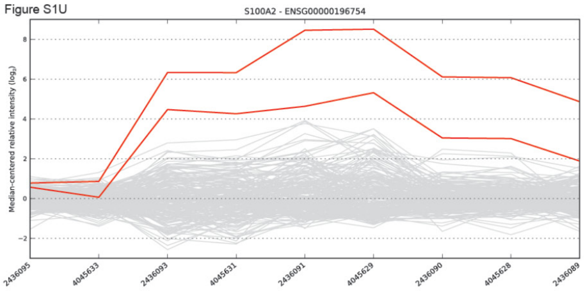

Figure S1V

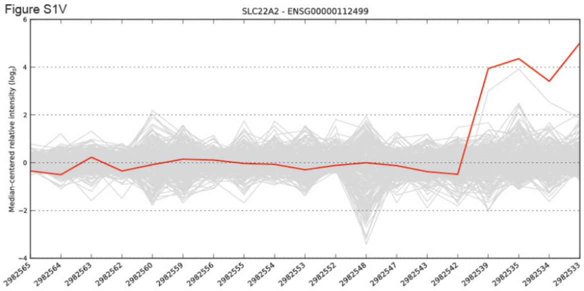

Figure S1W

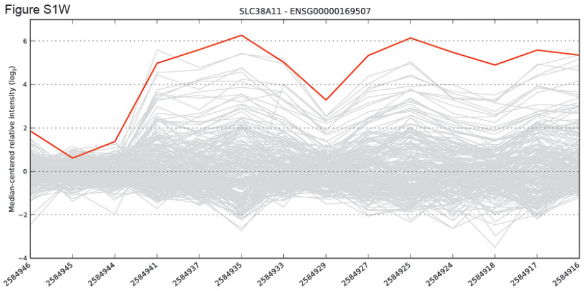

Figure S1X

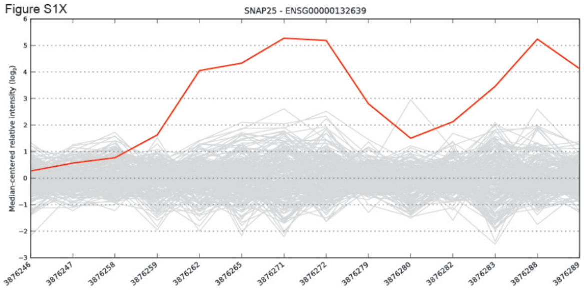

Figure S1Y

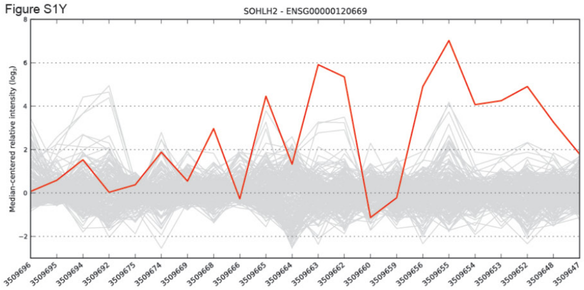

Supplementary Figure S1: Exon-level expression profiles for all 25 selected candidate genes. Each line represents an individual tumor sample, with the expression values median-centered and the most significantly deviating sample(s) marked in red. X-axis numbering refers to probe sets on the Affymetrix HuEx-1_0-st-v2 microarray.

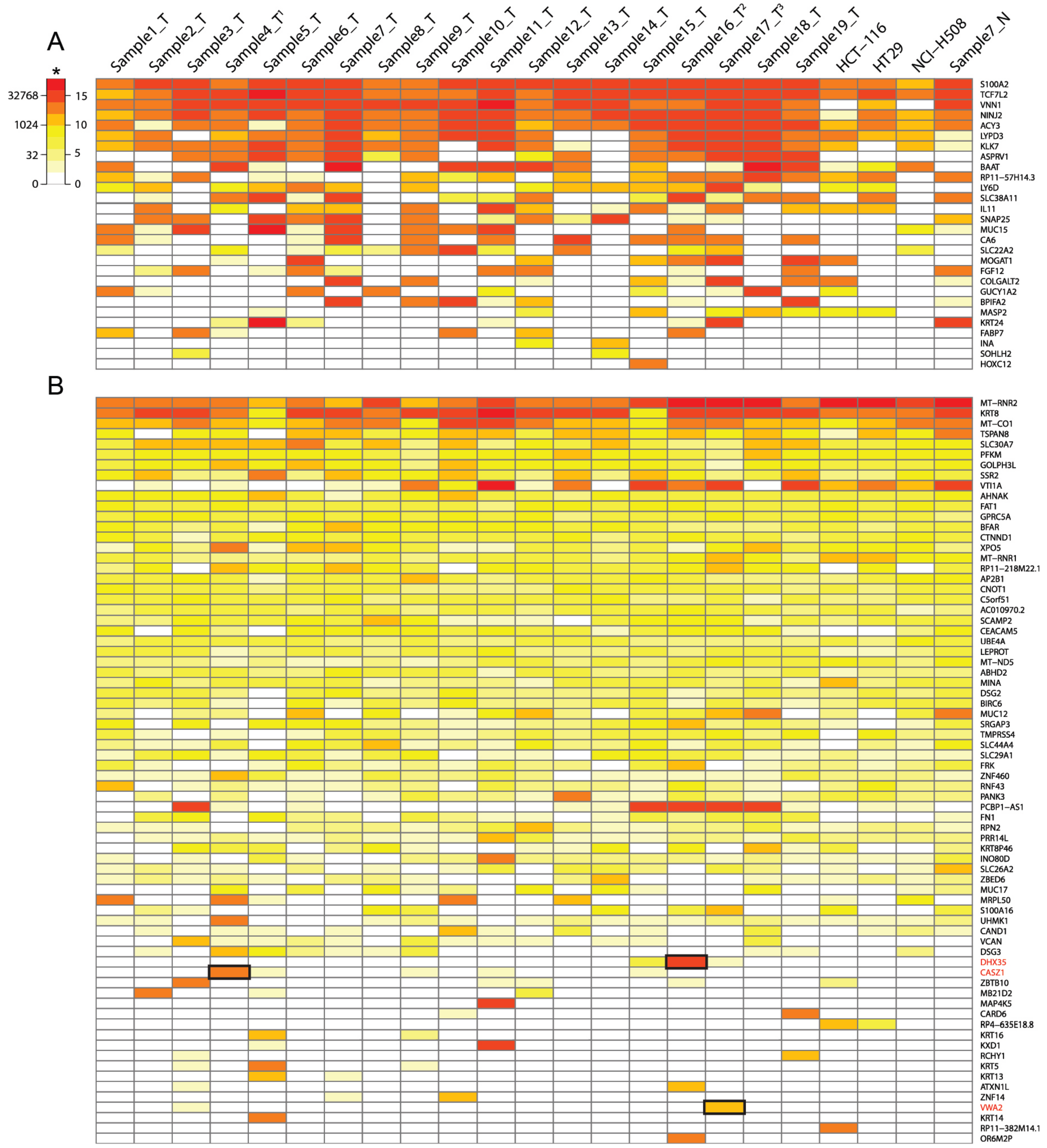

Supplementary Figure S2: Heat map showing log2 values of normalized read counts of the top 100 covered genes from the RACE-seq experiment. *Heat colors represent log2 values of normalized read counts. Corresponding read count values are also indicated. [1]Sample4_T was the index sample with elevated expression at the 3′ end of *MASP2*, as seen from the exon microarray data. [2]Sample16_T was the index sample with elevated expression at the 3′ end of *BPIFA2*, as seen from the exon microarray data. [3]A fusion between *VWA2* and *TCF7L2* was identified by defuse in sample17_T. The heat map is divided into **A.** Candidate genes targeted by RACE amplification, and **B.** top non-target genes. Upstream partner genes of the identified fusion transcripts *VWA2-TCF7L2, DHX35-BPIFA2* and *CASZ1-MASP2* are indicated with red font. Black boxes indicate the normalized read counts values in the samples expressing the identified fusion transcripts. For *DHX35* and *CASZ1*, the samples expressing the fusion transcripts were also the samples identified to have elevated 3′ expression as seen from the exon microarray data.

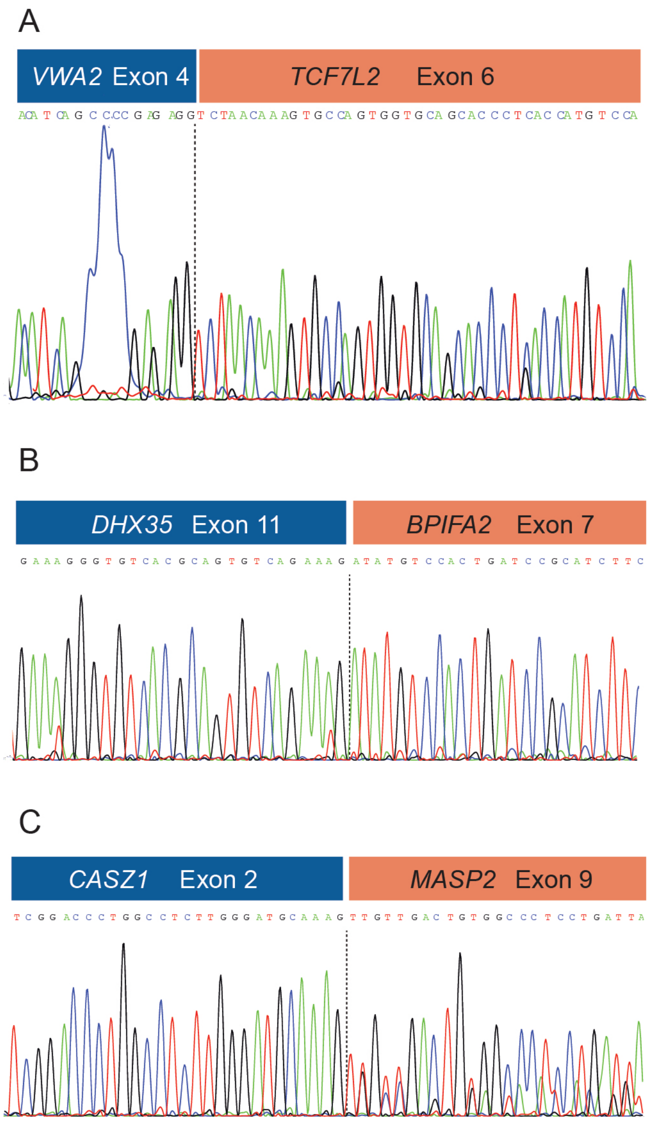

Supplementary Figure S3: Electropherograms from Sanger sequencing showing sequences covering intact exon to exon breakpoint boundaries for A. *VWA2-TCF7L2,* **B.** *DHX35-BPIFA2* and **C.** *CASZ1-MASP2*

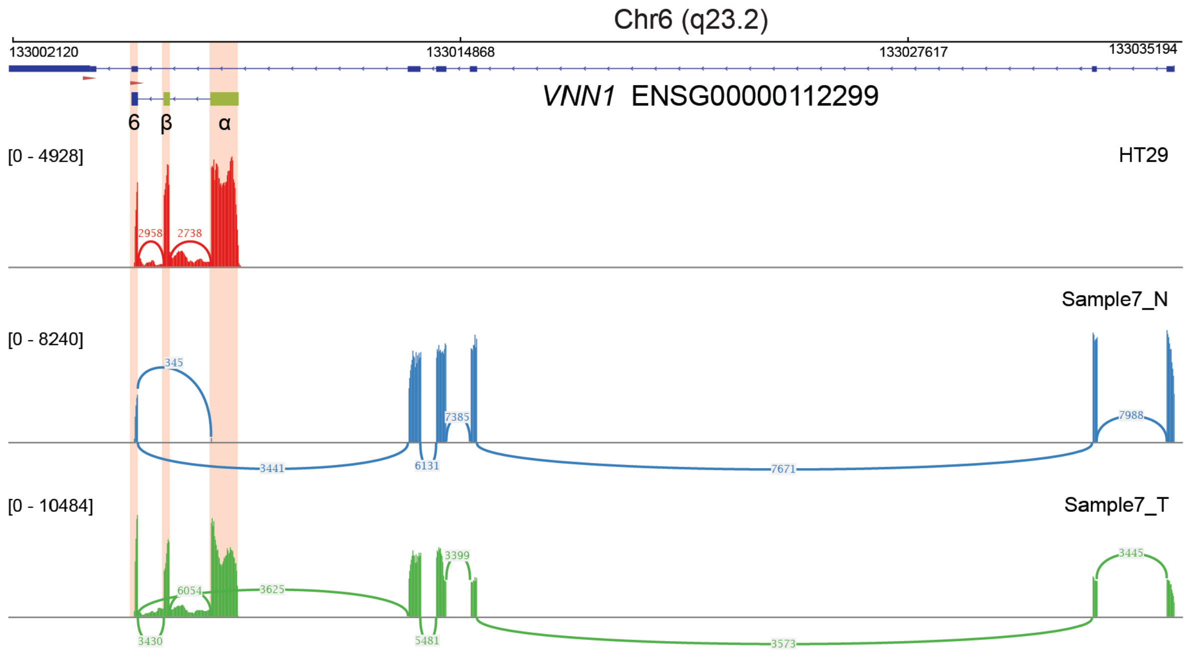

Supplementary Figure S4: Sashimi plot that shows the RACE-seq read coverage of the *VNN1* gene in the HT29 CRC cell line and the tumor/normal pair included in the experimental set up. Canonical UCSC gene annotation is shown on the top track, with red arrows indicating the location of the *VNN1* RACE assay. The alternative start exons α and β of the recently described transcript [22] are shown in green. Bars show coverage values at genomic locations, while arcs depict splicing junctions. The numbers of reads crossing the splicing junctions are annotated on the arcs, here determined by Tophat2 alignment and the sashimi plot package in IGV.

Supplementary Table S1: Sequence reads produced by high-throughput sequencing of RACE products, and alignment numbers before and after trimming.

Supplementary Table S2: Novel transcript splice junctions covered by a minimum of 100 reads from the RACE-seq data.

Supplementary Table S3: Transcript splice junctions used as input to the STAR aligner.

Supplementary Table S4: Number of aligned reads to input junctions, as determined by the STAR aligner.

Supplementary Table S5: Primers used in the RACE amplification of target genes.

Supplementary Table S6: RNA-seq data from The Cancer Genome Atlas, used for validation of fusion transcripts and splice junctions.

Supplementary Table S7: RNA-seq data from The Cancer Cell Line Encyclopedia used for validation of fusion transcripts and splice junctions.
